# Analysis of factors associated with operative difficulty in thoracoscopic esophageal cancer surgery in the left-decubitus position: a single-center retrospective study

**DOI:** 10.1186/s12893-023-02131-2

**Published:** 2023-08-18

**Authors:** Koichi Okamoto, Noriyuki Inaki, Hiroto Saito, Mari Shimada, Takahisa Yamaguchi, Toshikatsu Tsuji, Hideki Moriyama, Jun Kinoshita, Isamu Makino, Keishi Nakamura, Hiroyuki Takamura, Itasu Ninomiya

**Affiliations:** 1 Department of Gastrointestinal Surgery, Graduate School of Medical Science, Kanazawa University, 13-1 Takara- machi, Kanazawa, 920-8641 Ishikawa Japan; 2 Department of General and Digestive Surgery, Kanazawa Medical University Hospital, 1-1 Daigaku, Uchinadamachi, Kahoku, 920-0293 Ishikawa Japan; 3 Department of Gastroenterological Surgery, Ishikawa Prefectural Central Hospital, 2-1 Kuratsukihigashi, Kanazawa, 920-8530 Ishikawa Japan; 4 Department of Hepato-Biliary-Pancreatic Surgery, Graduate School of Medical Science, Kanazawa University, 13- 1 Takara-machi, Kanazawa, 920-8641 Ishikawa Japan; 5 Department of Surgery, Fukui Prefectural Hospital, 2-8-1 Yotsui, Fukui, 910-0846 Japan

**Keywords:** Esophageal cancer, Thoracoscopic surgery, Minimally invasive esophagectomy, Difficulty, Complication

## Abstract

**Background:**

The degree of difficulty in the overall procedure and forceps handling encountered by surgeons is greatly influenced by the positional relationship of intrathoracic organs in minimally invasive esophagectomy. This study aimed to identify the anatomical factors associated with the difficulty of minimally invasive esophagectomy assessed by intraoperative injuries and postoperative outcomes.

**Methods:**

Minimally invasive esophagectomy in the left-decubitus position was performed in 258 patients. We defined α (mm) as the anteroposterior distance between the front of the vertebral body and aorta, β (mm) as the distance between the center of the vertebral body and center of the aorta, and γ (degree) as the angle formed at surgeon’s right-hand port site by insertion of lines from the front of aorta and from the front of vertebrae in the computed tomography slice at the operator’s right-hand forceps hole level. We retrospectively analyzed the correlations among clinico-anatomical factors, surgeon- or assistant-caused intraoperative organ injuries, and postoperative complications.

**Results:**

Intraoperative injuries significantly correlated with shorter α (0.2 vs. 3.9), longer β (33.0 vs. 30.5), smaller γ (3.0 vs. 4.3), R1 resection (18.5% vs. 8.3%), and the presence of intrathoracic adhesion (46% vs. 26%) compared with the non-injured group. Division of the median values into two groups showed that shorter α and smaller γ were significantly associated with organ injury. Longer β was significantly associated with postoperative tachycardia onset, respiratory complications, and mediastinal recurrence. Furthermore, the occurrence of intraoperative injuries was significantly associated with the onset of postoperative pulmonary complications.

**Conclusions:**

Intrathoracic anatomical features greatly affected the procedural difficulty of minimally invasive esophagectomy, suggesting that preoperative computed tomography simulation and appropriate port settings may improve surgical outcomes.

## Introduction

Esophagectomy is thought to be effective in eliminating the cancerous tissue in esophageal cancer and mediastinal lymph nodes, which need to be dissected, but it is a highly invasive surgical procedure that is associated with high incidences of postoperative complications and mortality [1, 2]. In recent years, minimally invasive esophagectomy (MIE) using thoracoscopy has been developed to reduce the surgical invasiveness and postoperative complications [3–5]. In MIE, a thorough and safe mediastinal dissection procedure is needed for curative resection of esophageal cancer. The ideal mediastinal lymph node dissection in MIE requires setting the left edge of the descending aortic wall as the left limit and the anterior surface of the aorta and vertebral body as the dorsal limit. Previously, thoracoscopic esophagectomy was started in the left-lateral decubitus position (LDP), as done in open esophagectomy. Generally, conversion to open thoracotomy is thought to be required if urgent intraoperative events occur, including bleeding, other organ injury, or if resectability by MIE is difficult [5]. In clinical practice, we have often experienced procedural difficulties and an increase in the intrathoracic operation time in MIE performed with patients in the LDP due to the positional relationship of intrathoracic organs. The degrees of difficulty of handling the operator’s forceps and of the overall technique of lymph node dissection are greatly influenced by the position of the vertebral body and aorta and the narrowness of the mediastinum in MIE. We hypothesized that intraoperative complications, such as bleeding and organ injury, can be reduced significantly by preoperative evaluation of intrathoracic anatomical features, which appear to be strongly related to intraoperative injuries. However, only a few reports have described the clinico-anatomical features that can be risk factors for intraoperative complications, such as unexpected bleeding and organ injury [6, 7]. The present study aimed to evaluate the effect of various clinico-anatomical factors on the degree of MIE procedural difficulty in the LDP and on intraoperative bleeding, organ injury, and postoperative complications.

## Materials and methods

### Patients

We enrolled 258 patients who underwent MIE in the LDP at Kanazawa University Hospital between January 2003 and June 2018. The exclusion criteria were patients who underwent two-stage esophagectomy or preoperative radiotherapy. All patients were staged according to the Union for International Cancer Control TNM staging, version 8 [8]. Data were collected and analyzed retrospectively. Our institutional ethics committee approved this research (Registry Number 1774).

### Surgical procedure for thoracoscopic esophagectomy

**Fig. 1:**
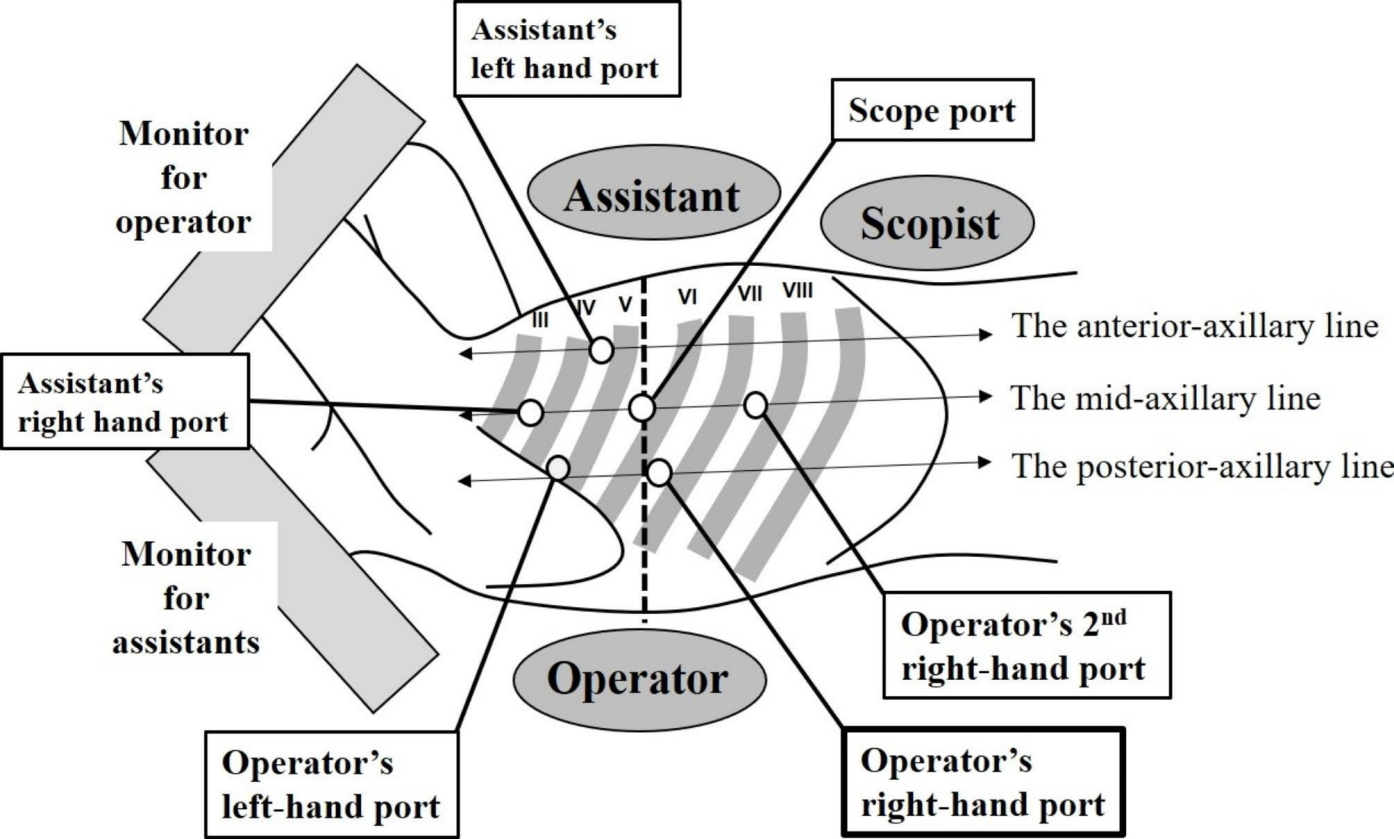
Schema showing the positions of the patient, operator, assistants, and trocar sites. Roman numerals indicate the rib numbers

MIE was basically performed as described previously [9]. During the thoracic procedure in the LDP, the patients were intubated with a double-lumen endotracheal tube or single-lumen endotracheal tube with a balloon blocker for one-lung ventilation. Ports setting and operating views are presented in Fig. 1. A forward-oblique viewing endoscope with a 30° down-facing orientation was used to create an operative view for the operator similar to that in open esophagectomy. The operator and assistants stood on the dorsal and ventral sides of the patient, respectively, as done in open esophagectomy. In the thoracic manipulation, a total of 6 ports are inserted. The ports for the use of operator’s right- and left-hand forceps were mainly inserted into the 6th and 4th intercostal spaces (ICSs) in the posterior axillary line, respectively. The port at the 7th ICS was used from the depending on the intraoperative situation. The right lung and trachea were retracted and compressed appropriately with an assistant’s tracheal retractor inserted from the 3rd and 4th ICSs in the anterior axillary line. Thoracoscopy was mainly inserted from the port at the 5th ICS in the mid-axillary line. In 59 cases, artificial pneumothorax with CO_2_ insufflation at a pressure of 8–12 mmHg was used. The operator used monopolar curved scissors and sealing devices for the tissue dissection. The image for the assistants was inverted horizontally and vertically. The Clavien–Dindo classification was used to categorize the surgical morbidities [10].

### Definition of the positional relationships among intrathoracic organs

In the first analysis, the patients were divided into two groups according to the presence or absence of intraoperative bleeding or organ injury caused by the operator’s or assistant’s manipulation with forceps, retractor, and/or energy devices. We defined the injured group as patients with injuries to the aorta (2 cases), azygous or intercostal vein (3 cases), lung (47 cases), and tracheobronchus (2 cases) which required repair. The patients’ characteristics, intraoperative factors, clinico-anatomical features retrospectively obtained from computed tomography (CT) imaging and postoperative complications were compared between the groups of 54 patients with intraoperative injury (injured group) and 204 patients without intraoperative injury (non-injured group).

**Fig. 2:**
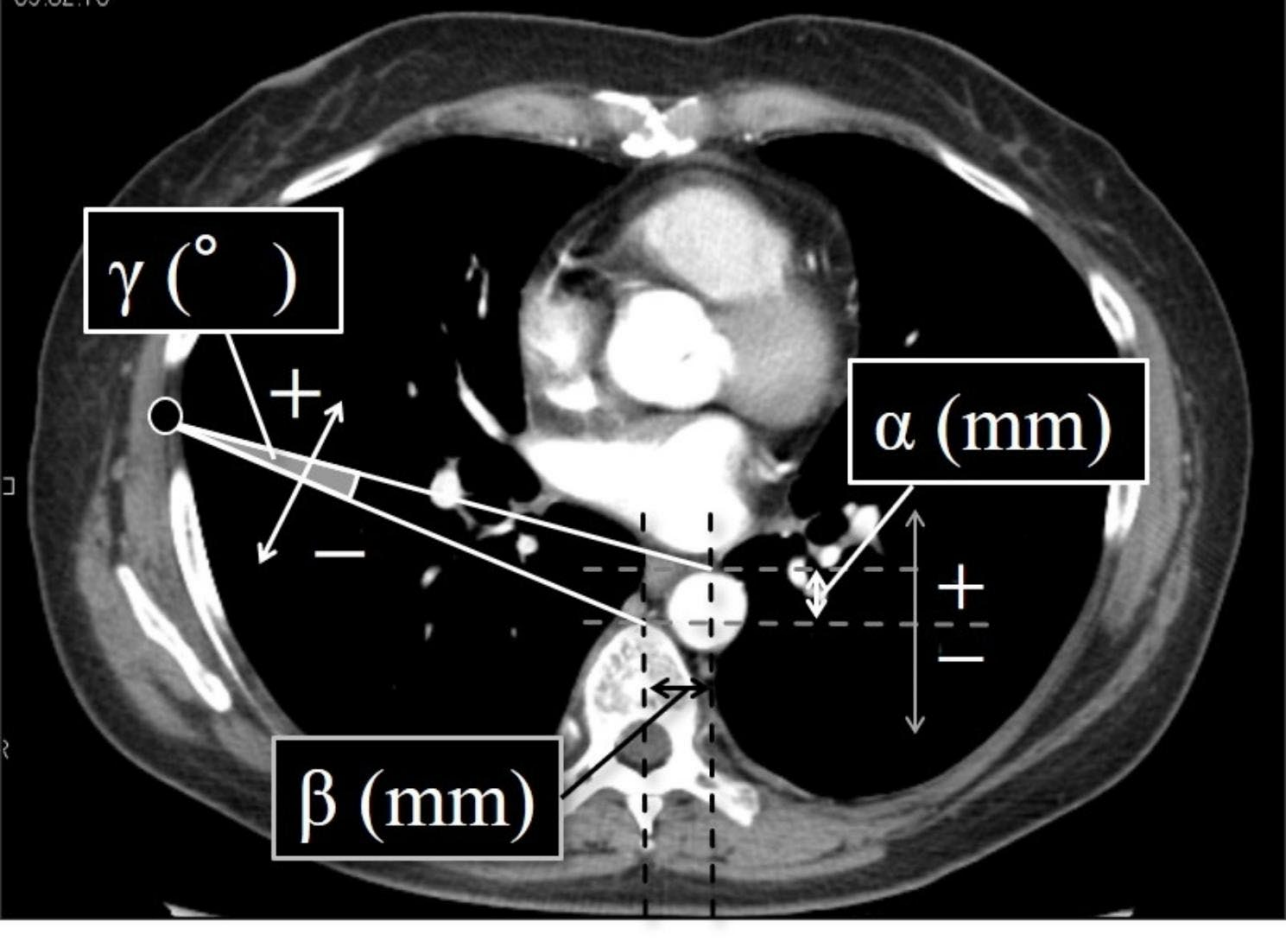
Definition of intrathoracic anatomical parameters. α (mm) = the anteroposterior distance between the front of the vertebral body and front of the aorta; β (mm) = the distance between the center of the vertebral body and center of the aorta; γ (°) = the angle formed at surgeon’s right-hand port site by insertion of lines from the front of the aorta and from the front of vertebrae

In the second analysis, we divided the patients into two groups according to their anatomical parameters at the operator’s right-hand port level determined from CT images obtained with the patients in the supine position. The schemes for the definition of each anatomical parameter are shown in Fig. 2. We used preoperative and postoperative CT images to define the following anatomical parameters at the operator’s right-hand port scar: α, β, and γ, where α (mm) = the anteroposterior distance between the front of the vertebral body and the front of the aorta, β (mm) = the distance between the center of the vertebral body and the center of the aorta, and γ (degree) = the angle between the front of the aorta-right-hand forceps and the front of the vertebral body. We retrospectively analyzed the correlations between the median values of each parameter with clinico-oncological features, intraoperative factors, and postoperative outcomes.

### Statistical analysis

Numeric results are expressed as the mean ± standard deviation. The χ^2^, Fisher’s exact, and Student’s *t*-tests were performed as appropriate to statistically analyze the clinico-anatomical variables that we feel may cause procedural difficulties in clinical practice and the incidences of postoperative complications. Statistical significance was assumed for *P* < 0.05. Some clinico-anatomical factors were selected for univariate and multivariate analyses of the risk factors for the incidence of postoperative complications. Factors with *P* < 0.05 were defined as independent risk factors for morbidity after MIE. All analyses were performed using SPSS (IBM SPSS Statistics, version 25; IBM Corp., Armonk, NY, USA).

## Results

### Correlations between clinico-anatomical features and intraoperative injuries

Table 1 shows the clinico-anatomical characteristics and intraoperative factors in the injured and non-injured groups. The port holes for the right-hand forceps of the operator were placed on the posterior axillary line at the level of the 5th to 7th ICS (5th : 6 cases, 6th : 193 cases, 7th : 59 cases). Comparison of each factor between both groups showed no differences in the clinico-oncological factors except age. On the other hand, there were significant differences in the correlations with the α value, β value, γ value, R1 resection rate, pleural adhesion, and intrathoracic operative time.

**Table 1:** Clinico-oncological characteristics, intraoperative factors, and anatomical parameters of the patients with or without intraoperative injury or bleeding

		Injured group (n = 54)	Non-injured group (n = 204)	*P* value
Age	Mean ± SD	68.0 ± 7.3	64.5 ± 8.1	*0.005 ^a^
Sex	male / female	47 / 7	161 / 43	0.180 ^b^
Performance status	0 / 1≦	47 / 7	193 / 11	0.052 ^b^
Tumor location	Upper / Middle / Lower	5 / 33 / 16	32 / 96 / 76	0.164 ^b^
Histology	SCC / Basaloid / AC / Others	50 / 1 / 2 / 1	178 / 5 / 14 / 7	0.745 ^b^
cT (UICC 8th )	is / 1 / 2 / 3 / 4	0 / 11 / 17 / 18 / 8	2 / 63 / 47 / 72 / 20	0.350 ^b^
cN (UICC 8th )	0 / 1 / 2 / 3	23 / 14 / 11 / 6	102 / 48 / 39 / 15	0.715 ^b^
cM (UICC 8th )	0 / 1	42 / 12	176 / 28	0.125 ^b^
cStage (UICC 8th )	0-I / II / III / IVA / IVB	10 / 16 / 6 / 10 / 12	63 / 53 / 42 / 19 / 27	0.039 ^b^
Emphysema	Present / Absent	14 / 40	46 / 158	0.601 ^b^
Height (cm)	Mean ± SD	164.3 ± 6.9	163.2 ± 9.9	0.345 ^a^
Body weight (kg)	Mean ± SD	57.9 ± 9.4	58.1 ± 9.9	0.871 ^a^
BMI	Mean ± SD	21.4 ± 3.0	21.7 ± 2.8	0.464 ^a^
Preoperative chemotherapy	Yes / No	29 / 25	93 / 111	0.288 ^b^
Total operation time (min)	Mean ± SD	673 ± 131	627 ± 119	*0.014 ^a^
Time of intrathoracic procedure (min)	Mean ± SD	343 ± 96	278 ± 70	*<0.001 ^a^
Total blood loss (g)	Mean ± SD	730 ± 973	563 ± 450	0.246 ^a^
Blood loss of intrathoracic procedure (g)	Mean ± SD	412 ± 858	233 ± 188	0.133 ^a^
No. of dissected mediastinal LN	Mean ± SD	34.4 ± 13.2	31.0 ± 12.1	0.070 ^a^
No. of mediastinal LN metastases	Mean ± SD	2.0 ± 5.0	1.2 ± 2.6	0.756 ^a^
Reconstruction route	Mediastinal / Retrosternal / Anterior	44 / 9 / 1	190 / 14 / 0	*0.011 ^b^
Artificial pneumothorax	Present / Absent	13 / 41	46 / 158	0.812 ^b^
Intrathoracic adhesion	Present / Absent	25 / 29	52 / 152	*0.003 ^b^
Resectability	R0 / R1≦	44 / 10	187 / 17	*0.030 ^b^
No. of intercostal port of right-hand forceps	5th / 6th / 7th	4 / 40 / 10	2 / 153 / 49	*0.017 ^b^
Distance of α (mm)	Mean ± SD	0.2 ± 10.3	3.9 ± 8.5	*0.007 ^a^
Distance of β (mm)	Mean ± SD	33.0 ± 6.7	30.5 ± 6.0	*0.008 ^a^
Degree of γ (°)	Mean ± SD	3.0 ± 3.4	4.3 ± 3.2	*0.009 ^a^

SD, standard deviation; SCC, squamous cell carcinoma; AC, adenocarcinoma; UICC 8th, the Union for International Cancer Control TNM staging, version 8; BMI, body mass index; LN, lymph node

*p < 0.05

^a^ Student’s *t*-test; ^b^ χ^2^ test

### Effect of intraoperative injury on the incidence of postoperative complications after MIE

Table 2 shows the incidence of postoperative complications after MIE. Pulmonary complications, including pneumonia and respiratory failure, were frequently observed in the injured group. There were no significant differences in other complications between the groups.

**Table 2:** Postoperative complications after MIE.

	Injured group (n = 54)	Non-injured group (n = 204)	*P* value
All complications (CD ≧ IIIa)	28 (51.9%)	113 (55.4%)	0.642 ^a^
Recurrent nerve palsy (CD ≧ IIIa)	4 (7.4%)	16 (7.8%)	> 0.999 ^b^
Atelectasis, sputum expectoration disorder (CD ≧ IIIa)	22 (40.7%)	80 (39.2%)	0.838 ^a^
Pneumonia (CD ≧ IIIa)	11 (20.4%)	20 (9.8%)	*0.034 ^a^
ARDS, respiratory failure (CD ≧ IIIa)	10 (18.5%)	16 (7.8%)	*0.020 ^a^
Pleural effusion (CD ≧ IIIa)	11 (20.4%)	51 (25.0%)	0.479 ^a^
Chylothorax (CD ≧ IIIa)	1 (1.9%)	4 (2.0%)	> 0.999 ^b^
Anastomotic leakage (CD ≧ IIIa)	9 (16.7%)	19 (9.3%)	0.122 ^a^
SSI (CD ≧ IIIa)	10 (18.5%)	36 (17.6%)	0.882 ^a^
ACS, heart failure (CD ≧ IIIa)	1 (1.9%)	4 (2.0%)	> 0.999 ^b^
Tachycardia (Paf, PSVT) (CD ≧ II)	12 (22.2%)	53 (26.0)	0.572 ^a^
Re-operation (CD ≧ IIIa)	2 (3.7%)	15 (7.4%)	0.538 ^b^
Mediastinal recurrence	9 (16.7%)	18 (8.8%)	0.094 ^a^

Numbers given as n (%). MIE, minimally invasive esophagectomy; CD, Clavien Dindo classification; ARDS, acute respiratory distressed syndrome; SSI, surgical site infection; ACS, acute coronary syndrome; Paf, paroxysmal fibrillation; PSVT, paroxysmal supraventricular tachycardia

^a^ χ^2^ test; ^b^ Fisher’s exact test

### Effect of defined anatomical variables on the incidence of intraoperative injuries

The median values of α, β, and γ values were + 3.95 mm, 31 mm, and + 4.2°, respectively. When the patients were divided into two groups according to each median value, the group with long α or large γ had fewer intraoperative injuries. The patients in the group with a long β median value had significantly more frequent postoperative complications, such as tachycardia and respiratory complications, than the group with a shorter β median value (Table 3). We experienced six (4.6%) cases involving conversion to open esophagectomy in the group with long α, short β, and large γ values; i.e., the cases with distant and shallow aorta (one case with severe adhesion, three cases with aortic injury, one case with an injury to the membranous bronchus wall, and 1 case that required reconstruction). The conversion rate to open thoracotomy was significantly lower in the group with short α and long β. In addition, the mediastinal lymph node or local recurrence rates were significantly higher in the group with a long β value.

**Table 3:** Effects of defined anatomical parameters on intraoperative injuries

		α ≧ 3.95 mm (n = 129)	α < 3.95 mm (n = 129)	P-value	β < 31 mm (n = 131)	β ≧ 31 mm (n = 127)	P-value	γ ≧ 4.2° (n = 131)	γ < 4.2° (n = 127)	P-value	
Intraoperative injury		17 (13.2%)	37 (28.7%)	*0.002 ^a^	22 (16.8%)	32 (25.2%)	0.097 ^a^	21 (16.0%)	33 (26.0%)	*0.049 ^a^	
Conversion to open thoracotomy		6 (4.7%)	0	*0.029 ^b^	6 (4.6%)	0	*0.030 ^b^	6 (4.6%)	0	*0.030 ^b^	
Atelectasis (CD ≧ IIIa)		47 (36.4%)	55 (42.6%)	0.308 ^a^	44 (33.6%)	58 (45.7%)	*0.047 ^a^	46 (35.1%)	56 (44.1%)	0.140 ^a^	
Left pleural effusion (CD ≧ IIIa)		28 (22.0%)	36 (27.9%)	0.279 ^a^	26 (20.2%)	38 (29.9%)	0.071 ^a^	30 (23.3%)	34 (26.8%)	0.516 ^a^	
Postoperative tachycardia (CD ≧ II)		31 (24.4%)	34 (27.0%)	0.639 ^a^	25 (19.5%)	40 (32.0%)	*0.023 ^a^	34 (26.6%)	31 (24.8%)	0.748 ^a^	
Mediastinal recurrence		12 (9.3%)	15 (11.6%)	0.542 ^a^	6 (4.6%)	21 (16.5%)	*0.002 ^a^	8 (6.1%)	19 (15.0%)	*0.020 ^a^	

Numbers given as n (%). CD, Clavien Dindo classification

^a^ χ^2^ test; ^b^ Fisher’s exact test

### Univariate and multivariate analyses of clinico-anatomical factors associated with postoperative complications

Univariate analyses showed that the presence of intrathoracic adhesion, retrosternal or anterior reconstruction route, R1 and more residual tumor, and an α ≤ 3.95 mm were significantly correlated with intraoperative injury. Multivariate analyses revealed that intrathoracic adhesion, retrosternal reconstruction route, and an α ≤ 3.95 mm were significant predictors of intraoperative injury (Table 4). In addition, it was shown that intraoperative injuries, including lung injury, are significant risk factors for postoperative respiratory complications in multivariate analysis (Table 5).

**Table 4:** Univariate and multivariate analyses of the risk factors for intraoperative injuries in MIE

Logistic regression analysis			Univariate analysis			Multivariate analysis		
			OR	95% CI	P-value	OR	95% CI	P-value
Sex		Female vs. Male	1.793	0.757–4.248	0.184			
Age		<70 y.o vs. ≧70 y.o	1.412	0.753–2.648	0.283			
Preoperative chemotherapy		Absent vs. Present	1.385	0.759–2.527	0.289			
Reconstruction route		Mediastinal vs. Retrosternal	3.086	1.285–7.407	0.012 *			
Abdominal procedure		Open vs. HALS	1.333	0.707–2.519	0.374			
Intrathoracic adhesion		Absent vs. Present	2.520	1.355–4.688	0.004 *	2.496	1.269–4.910	0.008 *
Resection of thoracic duct		Absent vs. Present	0.827	0.399–1.717	0.611			
Artificial pneumothorax		Present vs. Absent	1.089	0.538–2.204	0.812			
cT		0–2 vs. 3,4	1.130	0.620–2.062	0.689			
cN		0 vs. 1≦	1.348	0.736–2.469	0.334			
cM		0 vs. 1	1.795	0.844–3.817	0.129			
cStage		0–2 vs. 3–4	1.420	0.778–2.591	0.254			
Resectability		R0 vs. R1≦	2.500	1.072–5.848	0.034 *			
Height		<164.3 cm vs. ≧ 164.3 cm	0.929	0.509–1.692	0.809			
Body weight		<57.2 kg vs. ≧ 57.2 kg	1.000	0.549–1.822	> 0.999			
BMI		<21.5 vs. ≧ 21.5	1.412	0.774–2.578	0.261			
Distance α		≧ 3.95 mm vs. <3.95 mm	2.653	1.401-5.000	0.003 *	2.833	1.121–7.143	0.028 *
Distance β		<31 mm vs. ≧ 31 mm	1.669	0.908–3.067	0.099			
Degree γ		≧ 4.2° vs. <4.2°	1.838	0.997–3.390	0.051			

MIE, minimally invasive esophagectomy; CI, confidential interval; HALS, hand assisted laparoscopic surgery; BMI, body mass index

*P < 0.05. Variables were adjusted for in the multivariable logistic regression model

**Table 5:** Univariate and multivariate analyses of the risk factors for postoperative pulmonary complications after MIE

Logistic regression analysis			Univariate analysis			Multivariate analysis		
			OR	95% CI	P-value	OR	95% CI	P-value
Sex		Female vs. Male	1.208	0.535–2.728	0.650			
Age		<70 y.o vs. ≧70 y.o	1.349	0.671–2.713	0.401			
Preoperative chemotherapy		Absent vs. Present	1.074	0.551–2.094	0.289			
Reconstruction route		Mediastinal vs. Retrosternal	1.580	0.551–4.525	0.395			
Abdominal procedure		HALS vs. Open	1.239	0.610–2.519	0.552			
Intrathoracic adhesion		Absent vs. Present	1.637	0.818–3.276	0.164			
Resection of thoracic duct		Absent vs. Present	0.722	0.328–1.591	0.419			
Artificial pneumothorax		Present vs. Absent	1.497	0.710–3.157	0.290			
cT		0–2 vs. 3,4	1.092	0.558–2.139	0.797			
cN		0 vs. 1≦	1.106	0.566–2.160	0.768	2.398	0.916–6.250	0.075
cStage		0–2 vs. 3–4	1.509	0.758–3.006	0.242	3.102	1.152–8.351	0.025*
Resectability		R0 vs. R1≦	1.600	0.603–4.237	0.345			
Distance α		≧ 3.95 mm vs. <3.95 mm	1.059	0.543–2.066	0.865			
Distance β		<31 mm vs. ≧ 31 mm	0.979	0.502–1.909	0.951			
Degree γ		≧ 4.2° vs. <4.2°	0.872	0.446–1.701	0.687			
Intraoperative injury		Absent vs. Present	2.294	1.105–4.766	0.026 *	2.413	1.145–5.084	0.021*

MIE, minimally invasive esophagectomy; CI, confidential interval; HALS, hand assisted laparoscopic surgery;

*P < 0.05 Variables were adjusted for in the multivariable logistic regression model

## Discussion

Our findings showed the importance of the positional relationships of intrathoracic organs with intraoperative injury and postoperative complications in MIE performed in the LDP. Previously, it has been reported that smoking history, comorbidities, operation time, and intraoperative blood loss are important factors associated with the postoperative complications after esophagectomy [11–14]. In recent years, MIE has been induced in many institutions and its effectiveness and less invasiveness have been reported worldwide [5, 14, 15]. In esophageal cancer surgery, thorough mediastinal lymph node dissection is essential, with the left wall of the aorta and left mediastinal pleura chosen as the left border and the anterior surface of the aorta and vertebral body chosen as the dorsal border [5]. However, in clinical practice, we have often experienced procedural difficulties and an increase in intrathoracic operation time in MIE performed in patients with a narrow mediastinal space. In cases with a narrow mediastinal space and deep aorta, our experience has shown that the external force needed to manipulate the surrounding organs often increases because of the difficulty in obtaining a good operative field that enables a thorough mediastinal lymph node dissection. Even if enough lung collapse is achieved by performing isolated lung ventilation during MIE in the LDP, the procedure is greatly affected by the anatomical position and movements of the intrathoracic organs due to the compression and traction performed by the assistant. Consequently, intraoperative direct injury to the lung surface and mediastinal tissues by the retractor or the operator’s forceps can occur.

Only a few reports have demonstrated a mathematical correlation between procedural difficulty and intrathoracic anatomical features. Fujiwara et al. and Okamura et al. reported technical difficulties in performing MIE caused by the position of the descending aorta or width of the mediastinal space, which were related to the ventral–dorsal distance between the sternum and vertebra [7, 16]. Uchihara et al. reported that a left-sided esophagus can increase the MIE procedural difficulty and postoperative incidence of morbidity in the LDP [6]. Especially in patients with a narrow mediastinum and deep esophagus, it is assumed that the quality of the surgical technique is greatly affected by the ability of the assistant to maintain a good view of the operative field of the mediastinum by manipulating the organs. Additionally, procedures involving the mediastinal organs or vessels that are performed in a poor operative field can cause organ injury or bleeding. Contusions of the lung surface and internal damage caused by excessive external force against the lung, trachea, and bronchus caused by a retractor are thought to cause injuries that can lead to postoperative pneumonia and sputum expectoration disorder. Therefore, it is suggested that minimizing compression and retraction manipulations may reduce respiratory complications. In fact, the α value, which is an indicator of deep aorta, and the β value, which is an indicator of distant aorta, are significantly associated with intraoperative organ injuries, so the importance of these anatomical indices cannot be ignored. We believe that the distant aorta indicates the distant periesophageal tissue to be dissected. We have performed MIE with artificial pneumothorax by CO_2_ insufflation to protect the right lung from the potential damage caused by the forceps and retractor. In our experience, intrathoracic CO_2_ insufflation itself is theoretically beneficial to make a satisfactory operative field and reduce intrathoracic blood loss and secondary organ injury in the LDP [9]. It has been reported that physical characteristics, including a left-sided esophagus and strong lordosis, can correlate with aging and cardiovascular or pulmonary comorbidities and the onset of pulmonary complications after esophagectomy [6, 12, 17, 18]. Okamura, et al. reported that there was a significant correlation between the intraoperative time with the tumor depth, the presence of NAC, thoracic duct resection, and intrathoracic blood loss [16]. In addition, Guo et al. have reported that unplanned intraoperative events such as bleeding, intrathoracic adhesions, and serious tumor invasion that could not be anticipated preoperatively could be risk factors of postoperative pulmonary complications and chylothorax [19]. And inflammation, local edema, and sclerotic changes in the mediastinum following neoadjuvant chemoradiotherapy can increase intraoperative tracheal injury [20]. In our experience, we have sometimes encountered procedural difficulty in highly advanced cases, but cT, cN, cM, cStage, the presence of NAC, and tumor resectability were not independent predictive factors of intraoperative organ injury. On the other hand, conversion to open thoracotomy was frequently observed in the patients with longer α and shorter β. This finding means that secondary injury itself is frequent in patients with a shallow and non-distant aorta. It is possible that forceps tend to stick into the aortic wall, so care should be given to the usage and vector of the right-hand forceps.

To minimize the influences of a deep and distant aorta, narrow mediastinum, and presence of the right lung, MIE in the prone position has become popular in recent years [21–23]. Otsubo et al. reported that MIE in the prone position is more effective for improving postoperative oxygenation and reducing pulmonary complications than MIE in the LDP [22]. In the prone position, even with double-lung ventilation, the heart, lung, and esophagus are pulled downward by gravity, and it is possible to maintain a better operative field view without the need for the assistant to retract the right lung and because blood pools to the bottom of the mediastinum [23, 24]. Operating on the dorsal side of patients may present several challenges due to poor ergonomics and an increased risk of intraoperative events. Alternatively, conducting the operation on the ventral side of the patient with a prone or semi-prone position might be more suitable for dissection of the mid-to-lower thoracic esophagus. In cases where dissection involves the recurrent laryngeal nerve, both dorsal and ventral stances should be considered viable options. In addition, the anteroposterior distance may be longer in the mediastinum than in the LDP. Higuchi et al. reported that it is possible to predict the intrathoracic procedural difficulty by performing a preoperative CT scan in the prone position [25].

Furthermore, robotic surgery technology has been recently introduced for esophageal cancer surgery and is gradually becoming popular worldwide [26, 27]. By making good use of the multi-joint function of robotic arms, it is possible for a surgeon to gently manipulate organs and tissues during mediastinal dissection and maintain the operative field by themselves and may alleviate the procedural difficulty of MIE. On the other hand, transmediastinal surgery can also resolve this problem. Yoshimura et al. reported that postoperative pneumonia was significantly decreased in transmediastinal esophagectomy relative to that in transthoracic esophagectomy (0% vs. 24.3%; *P* = 0.008) [28]. Compared with transthoracic esophagectomy, transmediastinal esophagectomy may place less stress on the thoracic organs and not be affected by deep and distant esophagectomy. It is suggested that advances in devices and surgical techniques may eventually contribute to further development of esophageal cancer surgery.

There were several limitations in this retrospective study because of several influential factors, such as the surgeons’ and assistants’ proficiency, the use of artificial pneumothorax, and the stage of esophageal cancer. In addition, the preoperative CT imaging was performed in the supine patient position, which differs from the actual patient position, such as the LDP or prone position. However, we believe that the anatomical features obtained from preoperative CT in the supine position undoubtedly reflect the degree of procedural difficulty of MIE. From our results of the present study, we suggest setting the position of the surgeon’s right-hand port more cranial or more ventral in cases with deep or distant aorta and narrow mediastinum in order to reduce the difficulty of surgical procedures of MIE. And we also suggest selecting the prone position and using the artificial pneumothorax to reduce the influences of the narrow mediastinum and the presence of the right lung in order.

## Conclusion

We emphasized the importance of the positional relationships, including the narrowness of the mediastinal, the distance of intrathoracic organs from the right chest wall, and the disturbance of the vertebral body and aorta, on the procedural difficulty in intrathoracic MIE. Additionally, we have provided novel points of consideration with applicability to preoperative simulation using CT imaging and appropriate port settings that can improve the surgical outcomes in both the LDP and prone positions.

## Data Availability

The datasets used and analyzed during the current study available from the corresponding author on reasonable request.
